# A Reference Genome Sequence for Giant Sequoia

**DOI:** 10.1534/g3.120.401612

**Published:** 2020-09-18

**Authors:** Alison D. Scott, Aleksey V. Zimin, Daniela Puiu, Rachael Workman, Monica Britton, Sumaira Zaman, Madison Caballero, Andrew C. Read, Adam J. Bogdanove, Emily Burns, Jill Wegrzyn, Winston Timp, Steven L. Salzberg, David B. Neale

**Affiliations:** * Department of Plant Sciences, University of California, Davis, CA 95616; † Center for Computational Biology, Whiting School of Engineering, Johns Hopkins University, Baltimore, MD 21211; ‡ Institute for Physical Sciences and Technology, University of Maryland, College Park, MD 20742; § Department of Biomedical Engineering, Johns Hopkins University, Baltimore, MD 21218; ** Bioinformatics Core, University of California, Davis, CA 95616; †† Department of Computer Science and Engineering, University of Connecticut, Storrs, CT 06269; ‡‡ Department of Molecular Biology and Genetics, Cornell University, Ithaca, NY 14850; §§ Plant Pathology and Plant-Microbe Biology Section, School of Integrative Plant Science, Cornell University, Ithaca, NY 14853; *** Save the Redwoods League, San Francisco, CA 94104; ††† Department of Ecology and Evolutionary Biology, University of Connecticut, Storrs, CT 06269; ‡‡‡ Departments of Computer Science and Biostatistics, Johns Hopkins University, Baltimore, MD 21218

**Keywords:** genome assembly, giant sequoia, *Sequoiadendron giganteum*, disease resistance genes, conifer, gymnosperm

## Abstract

The giant sequoia (*Sequoiadendron giganteum*) of California are massive, long-lived trees that grow along the U.S. Sierra Nevada mountains. Genomic data are limited in giant sequoia and producing a reference genome sequence has been an important goal to allow marker development for restoration and management. Using deep-coverage Illumina and Oxford Nanopore sequencing, combined with Dovetail chromosome conformation capture libraries, the genome was assembled into eleven chromosome-scale scaffolds containing 8.125 Gbp of sequence. Iso-Seq transcripts, assembled from three distinct tissues, was used as evidence to annotate a total of 41,632 protein-coding genes. The genome was found to contain, distributed unevenly across all 11 chromosomes and in 63 orthogroups, over 900 complete or partial predicted NLR genes, of which 375 are supported by annotation derived from protein evidence and gene modeling. This giant sequoia reference genome sequence represents the first genome sequenced in the Cupressaceae family, and lays a foundation for using genomic tools to aid in giant sequoia conservation and management.

Giant sequoia, *Sequoiadendron giganteum* (Lindl.) J.Buchh., is a California endemic conifer found in fragmented groves throughout the U.S. Sierra Nevada mountain range. Giant sequoias are known for their substantial size; individual specimens can reach over 90 m in height, more than 10 m in diameter, and may exceed 1000 m^3^ of wood volume (Sillett *et al.* 2015). In addition to their considerable proportions, giant sequoias are among the oldest tree species, as individuals can live for over 3,200 years (Douglass 1919). Giant sequoia is one of the two redwood species in California, where it shares the title of state tree with sister species coast redwood (*Sequoia sempervirens* Endl.).

Though they have occupied their current range for millennia and were known by indigenous people for centuries before colonizers arrived, giant sequoias became icons of the American west beginning with the exploitation of the Discovery Tree in 1853 (Cook 1942). Despite the brittle nature of their wood, historical research indicates a third of groves were either completely or partially logged (Elliott-Fisk *et al.* 1996, cited by Burns *et al.* 2018). Giant sequoias were first protected in 1864 (Cook 1942), and have remained a cornerstone of the American conservation movement ever since.

While the majority (98%) of remaining giant sequoia groves are now protected (Burns *et al.* 2018), the species is listed as endangered (IUCN 2020) and is overall experiencing a decline (Schmid and Farjon 2013). The dwindling numbers of giant sequoia are largely attributed to a lack of reproductive success due in part to fire suppression over the last century (Stephenson 1994), as giant sequoia trees rely on extreme heat to open their cones and release seeds in addition to preparing the understory for germination. Though mature giant sequoias in natural stands appear to withstand most pests and diseases, recent research suggests giant sequoias are potentially susceptible to bark beetles, which can exacerbate the impacts of drought (Stephenson *et al.* 2018).

In plants, disease resistance is typically conferred by genes encoding nucleotide binding leucine-rich repeat (NLR) proteins that individually mediate responses to different pathogens. Recent work in *Pinus flexilis* showed that NLR genes co-localize with mapped disease resistance loci (Liu *et al.* 2019). In crop species, NLR genes also have demonstrated contributions to resistance against insects (Stahl *et al.* 2018). A recent examination of transcriptome data from several conifer species showed that many conifer NLR genes are downregulated in response to drought (Van Ghelder *et al.* 2019), suggesting contrasting roles in biotic *vs.* abiotic stress responses. Cataloging NLR genes in giant sequoia is a step toward understanding their impact in relation to conservation and management. Notably, however, across species and even among plant populations, NLR genes account for the majority of copy-number and presence/absence polymorphisms (Yu *et al.* 2011; Zheng *et al.* 2011; Xu *et al.* 2012; Bush *et al.* 2014; Schatz *et al.* 2014). This complexity makes accurate inventory challenging in the absence of a high-quality genome assembly.

More broadly, a whole genome reference assembly provides a foundation for understanding the distribution of genetic variation in a species, which is critical for conservation and management. Though studies of population genetics and phylogenetics of giant sequoia have been conducted using isozymes, microsatellites, RADseq, and transcriptomic data (Fins and Libby 1982; DeSilva and Dodd 2014; Dodd and DeSilva 2016; Scott *et al.* 2016) there is a dearth of robust genomic resources in this species. The closest species’ with fully sequenced genomes exist entirely in the family Pinaceae, which last shared a common ancestor with giant sequoia (Cupressaceae) more than 300 million years ago (Leslie *et al.* 2018).

A combination of short-read Illumina data, long-read Oxford Nanopore data, and Dovetail proximity ligation libraries produced a highly contiguous assembly with chromosome-scale scaffolds, many of which are telomere-to-telomere. This assembly also includes the largest scaffolds assembled to date in any organism. As a demonstration of the utility of the assembly, we undertook an initial examination of the number, distribution, and relationships of NLR genes. The giant sequoia genome assembly and annotation presented here is an unprecedented resource in conifer genomics, both for the quality of the assembly and because it represents an understudied branch of the gymnosperm tree of life.

## Materials and methods

### General sequencing, assembly, and annotation strategy

A combination of short-read Illumina sequence from haploid seed megagametophyte DNA, long-read Oxford Nanopore sequence from diploid needle DNA, and Dovetail proximity ligation libraries were generated from a giant sequoia tree, SEGI21. Genome assembly involved two major steps: contig assembly using a combination of short Illumina reads and very long Oxford Nanopore reads, and scaffolding with Hi-C libraries to provide long-range contiguity. The structural and functional annotation leveraged Iso-Seq transcripts from three tissues and a combination of informatic approaches to generate high quality protein-coding gene models.

### Sequencing and assembly

#### Megagametophyte DNA extraction and sequencing:

Cones were collected from a 1,360-year-old giant sequoia (SEGI21, Sillett *et al.* 2015) in Sequoia/Kings Canyon National Park in 2012. As in previous conifer genome sequencing projects (*e.g.*, Nystedt *et al.* 2013 and Zimin *et al.* 2014), the megagametophyte from a single fertilized seed was dissected out and its haploid DNA extracted with a Qiagen DNeasy Plant Kit (Hilden, Germany), followed by library preparation with an Illumina TruSeq Nano kit (San Diego, CA) using the low throughput protocol. This megagametophyte library was then sequenced on 10 lanes of an Illumina HiSeq 4000 (San Diego, CA) with 150 bp paired-end reads at the UC Davis Genome Center DNA Technologies Core facility.

#### Foliage DNA extraction and Nanopore sequencing:

In 2017 foliage was collected from the upper canopy of the same giant sequoia tree (SEGI21). From this foliage, high molecular weight DNA was extracted following the protocol developed by Workman *et al.* (2018). Briefly, purified genomic DNA was isolated through a nuclei extraction and lysis protocol. First, mature leaf tissue was homogenized in liquid nitrogen until well-ground, then added to a gentle lysis buffer (after Zhang *et al.* 2016, containing spermine, spermidine, triton, and β-mercaptoethanol) and stirred at 4° for ten minutes. Cellular homogenate was filtered through five layers of Miracloth into a 50mL Falcon tube, then centrifuged at 4° for 20 min at 1900 × g, which was selected based on the estimated giant sequoia genome size of around 9 Gb (Zhang *et al.* 2012; Hizume *et al.* 2001). Extracted nuclei were then lysed and gDNA precipitated using the Circulomics Nanobind Plant Nuclei Big DNA kit - alpha version (SKU NB-900-801-01). Then 1 μg of purified genomic DNA was input into the Ligation sequencing kit (LSK108-LSK109, Oxford Nanopore), according to protocol, with the exception of end repair optimization (100 μL sample, 14 μL enzyme, 6 μL enzyme at 20° for 20 min, then 65° for 20 min). Samples were sequenced on R9.4 minION flowcells using either the minION or GridION (Oxford Nanopore Technologies, Oxford, UK) for 48 hr, then raw fast5 data were basecalled with Albacore version 2.13 (Oxford Nanopore Technologies, Oxford, UK).

#### Hi-C and Chicago library preparation and sequencing:

Additional foliage from SEGI21 was submitted to Dovetail Genomics (Scotts Valley, CA) for Hi-C and Chicago library preparation as described by Putnam *et al.* 2016. Hi-C libraries preserve *in vivo* chromatin structures while Chicago libraries are based on *in vitro* reconstituted chromatin; the combination of these two approaches allows for marked improvement in contiguity for genome assemblies. Three Hi-C libraries and two Chicago libraries passed QC for sequencing and were sent to the UC San Francisco Center for Advanced Technology where they were pooled and sequenced on an Illumina Novaseq 6000 (San Diego, CA) in a single lane of an S4 flowcell (PE 150 bp).

#### Genome assembly:

Prior to assembly, genome size was estimated by counting 31-mers (all subsequences of 31 bases) in the Illumina reads and computing the histogram of the kmer frequencies *vs.* counts using jellyfish version 2.0 (Marçais and Kingsford 2011).

**Figure 1:**
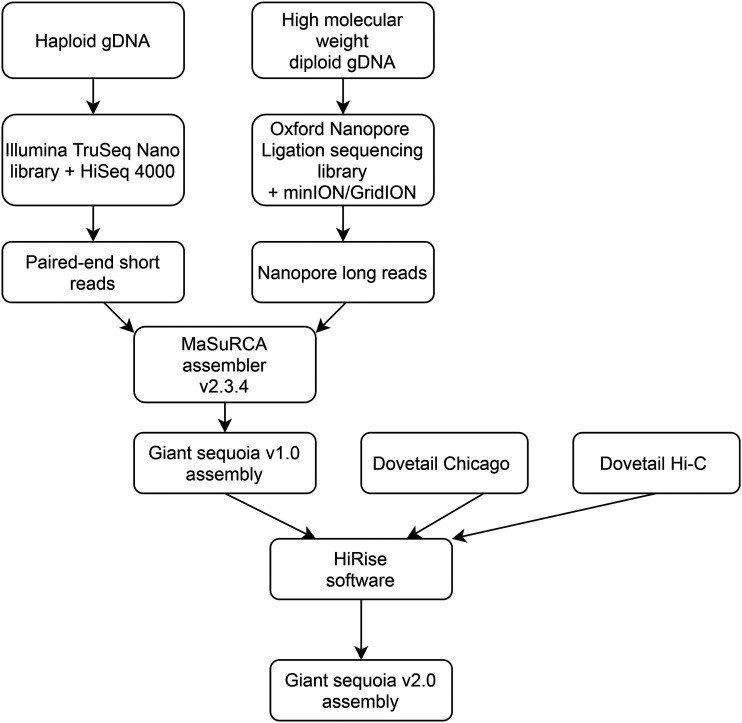
Flowchart of inputs and processing steps contributing to the giant sequoia v2.0 assembly.

Assembly of the giant sequoia genome (Figure 1) involved two major steps: contig assembly using Illumina and Oxford Nanopore reads, and scaffolding with “Chicago” and Hi-C libraries produced by Dovetail Genomics. Contigs were produced using MaSuRCA assembler version 3.2.4 (Zimin *et al.* 2013, Zimin *et al.* 2017) with the default parameters. The consensus error rate for the assembly was estimated by aligning the Illumina reads to the contigs with bwa-mem (Li 2013) and then calling variants with the Freebayes (Garrison and Marth 2012) software. Any site in the consensus that had no Illumina reads agreeing with the consensus and at least three Illumina reads agreeing on an alternative variant was considered an error. The total number of bases in the error variants were counted and divided by the total number of bases in the contigs. The initial contig assembly from MaSuRCA became version 1.0 and provided the foundation for downstream scaffolding.

We identified chloroplast contig based on their much-deeper coverage and their similarity to other chloroplast genomes. We then re-assembled the reads for these contigs and successfully assembled the chloroplast into a single, gap-free molecule of length 131,478 bp. We submitted the chloroplast as a separate entry in GenBank, where it has accession number CM017437. We separately aligned all contigs against a database of vectors and bacteria and removed any contigs that matched as presumed contaminants.

Sequence data from two Chicago libraries were used to scaffold giant sequoia 1.0 using Dovetail’s HiRise software (Putnam *et al.* 2016). Following this step, the output assembly comprised of Illumina, Oxford Nanopore, and Chicago data plus the Hi-C data were used as input for a second run of HiRise re-scaffolding software. The final scaffolded assembly was named giant sequoia 2.0.

#### Identification of centromeric and telomeric repeats:

Tandem repeat elements up to 500 bp long were identified with the tandem repeat finder program (trf v4.09; Benson 1999) with the recommended parameters (matching weight 2, mismatching penalty 7, indel penalty (delta) 7, match probability (PM) 80, indel probability (PI) 10, minimum alignment score to report (minscore) 50, maximum period size to report (maxperiod) 500). A histogram of repeat unit lengths was then produced, which had peaks at 7, 181, and 359 bp.

### Annotation

#### RNA isolation and sequencing:

Foliage and cambium were collected from a giant sequoia at Foresthill Divide Seed Orchard and immediately cooled in liquid nitrogen, then stored at -80° until extraction. Fresh root tissue was collected from a giant sequoia clone at the UC Davis Vegetable Crops greenhouse, stored in liquid nitrogen, and immediately ground for RNA extraction. RNA was isolated from the giant sequoia roots, foliage, and cambium using a LiCl-Urea buffer followed by cleanup using Zymo columns and reagents (Zymo Research, Irvine, CA). RNA quality was assessed using an Experion Electrophoresis System (Bio-Rad, Hercules, CA) and Qubit fluorometer (Thermo Fisher Scientific, Waltham, MA).

Double-stranded cDNA was generated from total RNA (2 µg per tissue) using the Lexogen TeloTM prime Full-length cDNA Kit (Lexogen, Inc., Greenland, NH, USA). Tissue-specific cDNAs were first barcoded by PCR (16-19 cycles) using IDT barcoded primers (Integrated DNA Technologies, Inc., Coralville, Iowa), and then bead-size selected with AMPure PB beads (two different size fractions of 1X and 0.4X). The three cDNAs were pooled in equimolar ratios and used to prepare a SMRTbell library using the PacBio Template Prep Kit (PacBio, Menlo Park, CA). The SMRTbell library was then sequenced on a Sequel v2 SMRT cell with polymerase 2.1 and chemistry 2.1 (P2.1C2.1) on one PacBio Sequel v2 SMRT cell at the UC Davis Genome Center DNA Technologies Core Facility.

#### Processing of IsoSeq data:

Raw IsoSeq subreads were processed using the PacBio IsoSeq3 v3.0 workflow (Töpfer 2019a, GitHub repository, https://github.com/PacificBiosciences/IsoSeq/). Briefly, ccs v.3.0.0 (Töpfer 2019b, GitHub repository, https://github.com/PacificBiosciences/ccs) was run to merge subreads one full-length circular consensus sequence (ccs) per Zero Mode Waveguide (ZMW). Then, lima v.1.7.0 (Töpfer 2019c, GitHub repository, https://github.com/PacificBiosciences/barcoding) was run to remove primer artifacts and to demultiplex the ccs by library barcode. Finally, isoseq3 cluster 3.0.0 was run to cluster the demultiplexed CCS reads into transcripts.

#### Repetitive element library generation and masking:

RepeatModeler (2.0; Smit *et al.* 2008) detected *de novo* repeats in the giant sequoia 2.0 assembly, after scaffolds shorter than 3 kbp were removed. The resulting repeat library, with classification, was used as input for RepeatMasker (v4.0.9, Smit *et al.* 2013) which soft masks repetitive elements in the genome. After this initial soft masking attempt, RepeatMasker was re-run with a library of conifer repeats identified in other gymnosperm species, clustered at 80%, to further mask repetitive elements.

#### Structural annotation:

PacBio IsoSeq transcripts and previously published Illumina RNAseq reads (Scott *et al.* 2016) were aligned to the soft masked genome, using Minimap2 v.2.12 (Li 2018) for the long-read data and HISAT2 v.2.1.0 (Kim *et al.* 2015) for short reads. The resulting alignment files were merged and sorted, then used alongside protein evidence generated with GenomeThreader (Gremme *et al.* 2005), provided as input to Braker2 v2.1.2 (Stanke *et al.* 2006; Stanke *et al.* 2008; Hoff *et al.* 2016; Hoff *et al.* 2019) to generate putative gene models.

#### Functional annotation:

Structural gene predictions were used as input for Eukaryotic Non-Model Transcriptome Annotation Pipeline (EnTAP; Hart *et al.* 2020), to add functional information and to identify improbable gene models. EnTAP was run in runP mode with taxon = Acrogymnospermae using the RefSeq Plant and SwissProt databases plus a custom conifer protein database (O’Leary *et al.* 2016; The Uniprot Consortium 2019). To further filter putative gene models, gFACs (Caballero and Wegrzyn 2019) was used, first by separating multiexonic and monoexonic models. Multiexonics were retained after filtering out models with non-canonical splice sites, micro-introns and micro-exons (<20 bp), and in-frame premature stop codons to ensure correct gene structure. Additionally, to control for function, genes annotating through Inteproscan (Jones *et al.* 2014) as retrodomains (including gag-polypeptide, retrotransposon, reverse transcriptase, *copia*, *gypsy*, and *ty1*) were discarded. In addition, any multiexonic models that lacked functional annotation, either with a sequence similarity hit or gene family assignment, were removed. Additionally, gffcompare (Pertea and Kirchner 2020, Pertea and Pertea 2020) identified overlap between gene models and softmasked regions of the genome, and multiexonic gene models were removed if more than 50% of the coding region was masked. Clustered transcriptome sequences were aligned to the genome using GMAP (v. 2018-07-04; Wu and Watanabe 2005; Wu and Nacu 2010) with a minimum trimmed coverage of 0.95 and a minimum identity of 0.95. To determine overlap and nesting of gene models with this high confidence transcriptomic alignment, BEDtools (Quinlan and Hall 2010). BUSCO v.4.0.2 (Simão *et al.* 2015) was used to assess the completeness of the filtered gene space. A figure summarizing these results was made in R version 3.6.3 (R Core Team 2020) using package karyoploteR (Gel and Serra 2017) installed with the BiocManager package (Morgan 2019).

#### Orthogroup assignment of proteins:

Translated UniGenes for all available gymnosperms were downloaded from the forest genomics database TreeGenes (https://treegenesdb.org/; Falk *et al.* 2018; Wegrzyn *et al.* 2019). The corresponding files from the *Amborella trichopoda* genome assembly (Amborella Genome Project 2013) were also included to provide an outgroup to the gymnosperm taxa (accessed via Ensembl, Howe *et al*. 2020). To create a nonredundant set of unigenes, transcripts and protein sequences were clustered with USEARCH (Edgar 2010) at 80% identity (Supplemental Figure S2). Each taxon with at least 15k unigenes was evaluated for completeness with BUSCO v4.0.2 (Simão *et al.* 2015) in protein mode using the Embryophyta lineage of OrthoDBv10 (Kriventseva *et al.* 2019). All taxa with at least 60% completeness were included in OrthoFinder (Emms and Kelly 2015; Emms and Kelly 2019) to identify orthogroups. For the purpose of functional annotation, the longest sequence from each orthogroup was retained, regardless of source species. Species-specific orthogroups unique to giant sequoia were noted. The resulting nonredundant species-specific orthogroups were functionally annotated with EnTAP in runP mode with taxon = *Sequoiadendron* using NCBI’s RefSeq Plant Protein and SwissProt databases.

#### Gene family evolution:

Following orthogroup assignment with OrthoFinder, a species tree and orthogroup statistics were used as input for CAFE v5 (Hahn *et al.* 2005; De Bie *et al.* 2006; Zenodo https://doi:10.5281/zenodo.3625141, as developed on GitHub) to assess gene family contraction and expansion dynamics, using a single birth/death parameter (λ) across the phylogeny. Figures summarizing the results were made using R version 3.6.3 (R Core Team 2020) using packages tidyverse (Wickham *et al.* 2019), tidytree (Yu 2020), ggplot2 (Wickham 2009), and ggtree (Yu *et al.* 2017; Yu *et al.* 2018). Gene families in the giant sequoia lineage experiencing rapid evolution were then functionally annotated using EnTAP.

#### Annotation and analysis of NLR genes:

NLR genes were identified using the NLR-Annotator pipeline (Steuernagel *et al.* 2018) on the giant sequoia 2.0 assembly, then that output was cross-referenced with the genome annotation. Using the genome annotation file and the NLR gene file as input, the BEDtools intersect function (Quinlan and Hall 2010) was used to identify putative NLR genes that were also present in the annotation, requiring features in the NLR gene file to overlap with 100% of the annotation feature. NLR-gene maximum likelihood trees were generated with RAxML v8.2.12 (Stamatakis 2014) using the amino acid sequence of the central NB-ARC domain output by NLR-Annotator. The DUMMY2 amino-acid substitution model was selected by running the -m PROTGAMMAAUTO option in RAxML. NB-ARC domains that included greater than 50% missing data were excluded from all analyses. The best trees were visualized with the Interactive Tree of Life (iTOL) tool, with bootstrap values shown (Letunic and Bork 2016). Determination of TIR and CC domains was based on motif data from Jupe and colleagues (2012). RPW8-like motifs were determined by alignment to a recently described RNL motif (CFLDLGxFP) (Van Ghelder *et al.* 2019).

### Data availability

The genome assembly of giant sequoia is available at NCBI under accession GCA_007115665.2, and raw sequence data are available under accessions SRX5827056 - SRX5827083. Annotation files are available at https://treegenesdb.org/FTP/Genomes/Segi. Supplemental material available at figshare: https://doi.org/10.25387/g3.12743378.

## Results and Discussion

### Sequencing and assembly

Assembly of the giant sequoia genome leveraged sequence data from four libraries (Supplementary Table S1). Illumina reads (135x) from a haploid megagametophyte library combined with Oxford Nanopore sequence from foliage (21x) contributed to the contig assembly. The contig assembly was subsequently scaffolded with data from Dovetail Chicago (47x) and Hi-C libraries (76x) in succession.

### Giant sequoia 1.0 assembly

Initial contig assembly of the Illumina and Oxford Nanopore sequence data yielded giant sequoia v1.0. Genome size was estimated by counting 31-mers (all sub-sequences of 31 bases) in the Illumina reads and computing the histogram of the kmer frequencies *vs.* counts using jellyfish tool version 2.0 (Marçais and Kingsford 2011). The histogram of 31-mer frequency counts had its largest peak at 101 (see Figure S1). There was a small second peak at 204, roughly double the highest 31-mer frequency of 101, likely corresponding to 2x repeat sequences in the genome. The k-mer coverage of the genome was then estimated by computing the area under the curve for frequencies between 1 and 10,000 and dividing that number by 101. This method arrived at the genome size estimate of 8,588 Gbp, consistent with the 9 Gbp estimate by Hizume *et al.* 2001.

The intermediate step of correction of the Nanopore reads in MaSuRCA resulted in 24,279,305 mega-reads with an average read length of 6,726 bp. The assembly error rate was calculated at 0.3 errors per 10000 bases, or consensus quality of 99.997%.

The resulting assembly, giant sequoia 1.0, had a contig N50 size of 347,954 bp and a scaffold N50 size of 490,521 bp.

### Giant sequoia 2.0 assembly

The Dovetail HiRise Chicago and Hi-C assembly increased the total assembly size marginally, to 8.125 Gbp, but notably yielded a large increase in the scaffold N50 size, to 690.6 Mb (Table 1). The overall number of scaffolds was reduced to 8,125, and the scaffold N90 size of the final assembly was 844.6 Mb. It is worth noting that the largest scaffold in this assembly is 985 Mbp in length, making it the longest contig assembled to date in any organism.

**Table 1:** Assembly statistics for the initial and final scaffolded assembly of giant sequoia

Assembly	Total sequence (bp)	N50 contig size (bp)	N50 scaffold size (bp)	Number of contigs	Number of scaffolds
Giant sequoia 1.0	8,122,145,191	347,954	490,521	49,651	39,821
Giant sequoia 2.0	8,125,622,286	347,954	690,549,816	52,886	8,215

The tandem repeat finder program (trf v4.09, G. Benson 1999) identified repeat elements up to 500 bp long, and those data were used to plot a histogram of repeat unit lengths which had peaks at 7, 181, and 359 bp. Based on the position and clustering along the chromosomes, the 7-mer was identified as the telomeric repeat and the 181-mer as the centromeric one.

The most common telomeric heptamers were TTTAGGG (found in most land plants, as reviewed by Peska and Garcia 2020), and TTGAGGG. The two heptamers alternate and have similar frequencies.

The 181 bp centromeric repeat unit consensus sequence was AAAAATTGGAGTTCGCGTGACACAGATGCAACGTAGCCTTAAAATCAGGTCTTCGCCGAACTCGACATTAAATCGATGGAAATTCAACATTCACGAAAACTGATAGAAAATAAAGGTTCTTAATAGTCATCTACAACACAATCTAAATCAAAGTTCTCCAAACATGGTTGATTATGGGTG.

By looking at the positions of the centromeric and telomeric repeats, a mis-assembly was identified in the original HiRise reference. Two centromeric and one telomeric region were located in the middle of the longest scaffold (1.82Gb), and subsequently this scaffold was split into chr1 (0.95Gb) and chr3 (0.84Gb).

There are 11 chromosomes in giant sequoia (Buchholz 1939; later confirmed by Jensen and Levan, 1941 and Schlarbaum and Tschuiya 1984), and the 11 largest scaffolds in the assembly span across the centromere (Table 2), suggesting a chromosome-level assembly. The 11 largest scaffolds range from 443 Mbp to 985 Mbp in size. Of these 11 scaffolds, seven include telomeric sequence on both ends. The remaining four scaffolds have telomeric sequence on one end. Beyond the 11 largest scaffolds, the next largest (Sc7zsyj_3574) (171 Mb) includes telomere at one end, suggesting it is a substantial portion of a chromosome arm for one of the scaffolds with only one telomere (chromosomes 1, 3, 6, and 9).

**Table 2:** Summary of largest scaffolds in giant sequoia 2.0

Scaffold ID	Length (bp)	Centromere?	Number of telomeres	Number of gaps	Total gap length (bp, estimated)
chr1	986,618,365	Y	1	4415	441,500
chr2	873,713,311	Y	2	3812	877,827
chr3	843,110,718	Y	1	3788	378,800
chr4	722,823,090	Y	2	3028	666,733
chr5	690,549,816	Y	2	2902	382,479
chr6	676,903,824	Y	1	3005	1,306,128
chr7	659,235,867	Y	2	2790	279,000
chr8	649,867,199	Y	2	2953	295,300
chr9	641,211,466	Y	1	2707	1,748,814
chr10	632,191,860	Y	2	2642	339,803
chr11	443,565,592	Y	2	1885	1,006,377
Sc7zsyj_3574	171,454,409	N	1	731	1,052,509

Summary of largest scaffolds in giant sequoia 2.0, showing that the 11 largest scaffolds represent near-complete chromosomes. All chromosomes other than these top 12 were less than 1 Mbp in length. Number of gaps and total gap length are shown in the final two columns; small gaps of unknown size were assigned a size of 100 bp. Where all gaps fell into this category, the total gap length is the number of gaps x 100.

### Assessing assembly completeness

For a rough estimate of the assembly completeness, BUSCO v4.0.2 (Simão *et al.* 2015) was run with the embryophyta lineage of OrthoDB 10 (Kriventseva *et al.* 2019) of 1614 genes. For the complete giant sequoia 2.0 genome, the tool found 612 complete BUSCOs out of which 576 were in a single copy, 36 were duplicated, and 192 were fragmented BUSCOs (Table 3). Another 810 BUSCOs were missing. In both the full giant sequoia 2.0 assembly and the version filtered to remove all scaffolds smaller than 3 kbp, completeness was estimated at 38% using BUSCO. Assembly completeness of other conifer assemblies (Supplementary Table S2) range from 27–44%, suggesting giant sequoia 2.0 completeness is consistent with existing work (*e.g.*, Nystedt *et al.* 2013; Zimin *et al.* 2014; Warren *et al.* 2015). Despite the contiguity of the assembly, the BUSCO completeness of the genome appears lower than expected, likely due to the presence of very long introns in conifers, which can inhibit identification of genes.

**Table 3:** BUSCO completeness of giant sequoia 2.0 assembly and annotation

	Giant sequoia v2.0	Giant sequoia v2.0 (≥3kbp)	Transcriptome	Transcriptome mapped to genome	High-confidence gene set
Number of input sequences	8215	8120	25859	22697	41633
Complete BUSCOs (C)	612	613	1377	1184	806
Complete and single-copy BUSCOs (S)	576	577	1333	1140	751
Complete and duplicated BUSCOs (D)	36	36	44	44	55
Fragmented BUSCOs (F)	192	191	95	84	260
Missing BUSCOs (M)	810	810	142	346	548
Total BUSCO groups searched	1614	1614	1614	1614	1614
Percentage found	37.92%	37.98%	85.32%	73.36%	49.94%

Completeness of giant sequoia 2.0 assembly and gene sets assessed with BUSCOv4.0.2. Giant sequoia v2.0 is the entire assembly and giant sequoia v2.0 (≥3kbp) only includes scaffolds at least 3kbp in length.

### Comparison to existing gymnosperm assemblies

The contiguity of giant sequoia 2.0 is most apparent when comparing with other gymnosperm assemblies (Table 4). Giant sequoia 2.0 has an N50 scaffold size of 690Mb, an order of magnitude larger than scaffold N50s reported in other conifers.

**Table 4:** Comparison of giant sequoia v2.0 assembly and annotation to selected gymnosperm genome projects

A	*Sequoiadendron giganteum*	*Abies alba*	*Picea glauca*	*Pinus lambertiana*	*Pinus taeda*	*Pseudotsuga menzesii*	*Ginkgo biloba*	*Gnetum montanum*
Reference		Mosca *et al.*, 2019	Warren *et al.*, 2015	Stevens *et al.*, 2016	Neale *et al.*, 2014	Neale *et al.*, 2017	Guan *et al.*, 2016	Wan *et al.*, 2018
Genome size (Mbp)	8,114	18,167	20,000	31,000	20,613	15,700	10,610	4,110
Chromosomes	11	12	12	12	12	12	12	22
TE content (%)	79	78	N/A	79	81	72	77	86
N50 scaffold size (kb)	690,549	14.05	71.50	246	107	340	1,360	475

B	*Sequoiadendron giganteum*	*Abies alba*	*Picea glauca*	*Pinus lambertiana*	*Pinus taeda*	*Pseudotsuga menzesii*	*Ginkgo biloba*	*Gnetum montanum*
Number of genes	37,936	94,209	14,462	38,518	51,751	46,688	41,840	27,493
Average overall CDS size (bp)	1,084	629	1,421	1,102	1,131	1,180	1,186	1,290
Average size introns (bp)	4,067	315	603	11,468	5,596	4,685	7,884	1,769
Maximum intron size length (kb)	1,399.11	36.01	119.32	1,254.69	758.52	351.90	1,272.92	342.13

Assembly (A) and annotation (B) statistics for giant sequoia v2.0 compared to recent gymnosperm genome projects. **A** Genome size, TE content, and N50 scaffold size are as reported in the literature. **B** Number of genes, average coding sequence (CDS) size, average intron size, and maximum intron length as calculated by gFACs.

### Annotation of giant sequoia 2.0

#### Repeat annotation:

Using the custom repeat database created by RepeatModeler, the majority (72.85%) of the giant sequoia genome was softmasked. Subsequent masking using conifer-specific repeat libraries yielded an additional 6% of masked sequence. LTRs were the most abundant known element (28%, Supplementary Table S3) in the masked sequence. These results are comparable to observations from different conifer species, *e.g.*, the most recent *Pinus lambertiana* assembly contained 79% repetitive sequence (Stevens *et al.* 2016). That our observations are consistent with the only conifer lineage sequenced until now (Pinaceae) is not surprising, as all conifers have large genome sizes, and this genomic bloat is attributed to the proliferation of repetitive elements throughout the genome (Neale *et al.* 2014).

#### Gene annotation:

Structural annotation using BRAKER2 resulted in 1,460,545 predicted gene models, with an average intron length of 2,362 bp (Table 5). The average coding sequence (CDS) length was 613 bp, including both multi and monoexonic models. The initial gene set included models with long introns, with the longest measuring 385,133 bp. The number of monoexonic genes (941,659) was almost twice as large as the total number of multiexonic gene models (518,886). Even with reasonable filters, the number of *ab initio* predicted monoexonic genes was highly inflated. Therefore, the monoexonic *ab initio* genes were removed from the gene space. The *ab initio* gene space was expanded by the addition of 14,538 well aligned unique transcriptome sequences of which 6,982 are monoexonic and the remaining 7,556 are multiexonic. After filtering, annotation yielded 41,632 high quality gene models. The average CDS length increased to 1,083 bp. The proportion of monoexonics (5,165) to multiexonics (36,466) was drastically reduced using the transcriptome as an evidence source. Long introns were maintained, with the maximum intron length in the high quality set reaching nearly 1.4 Mb.

Of the 41,632 high quality gene models, 35,183 were functionally annotated by either sequence similarity search or gene family assignment with EnTAP. These functionally annotated gene models include the longest plant intron found so far, at 1.4 Mb. Large introns are characteristic of conifer genomes, with introns up to 800 Kbp observed in *Pinus taeda* (Wegrzyn *et al.* 2014) and introns over 500 Kbp in *Pinus lambertiana* (Stevens *et al.* 2016).

**Table 5:** Gene models proposed by BRAKER2, before and after filtering

	Initial model set	Intermediate filtered set	High-confidence set
Total Genes	1,460,545	32,360	41,632
Average CDS length (bp)	613.90	1099.08	1146.4
Average number of exons	2.78	4.22	4.48
Average intron length (bp)	2,362	2,233	3,894
Max intron length (bp)	385,133	159,979	1,399,110
Total monoexonics	941,659	—	5,165
Total multiexonics	518,886	32,360	36,466

Intermediate set was filtered by removing monoexonic models, models with greater than 50% of their length in a masked region, models annotated as retrodomains, and models lacking functional annotation with EnTAP. The high-confidence set includes the intermediate set, plus monoxonic and multiexonic models derived from transcript evidence, removing any fully nested gene models.

Functional annotation of the gene containing the 1.4 Mb long intron suggests it is a member of the WASP (Wiskott-Aldrich syndrome protein) family. Wiskott-Aldrich syndrome proteins are in turn members of the SCAR/WAVE (suppressor of cAMP receptor/WASP family verprolin homologous) gene regulatory complex, which in plants has an important role in cell morphogenesis via activation of actin filament proteins (Yanagisawa *et al.* 2013).

**Figure 2:**
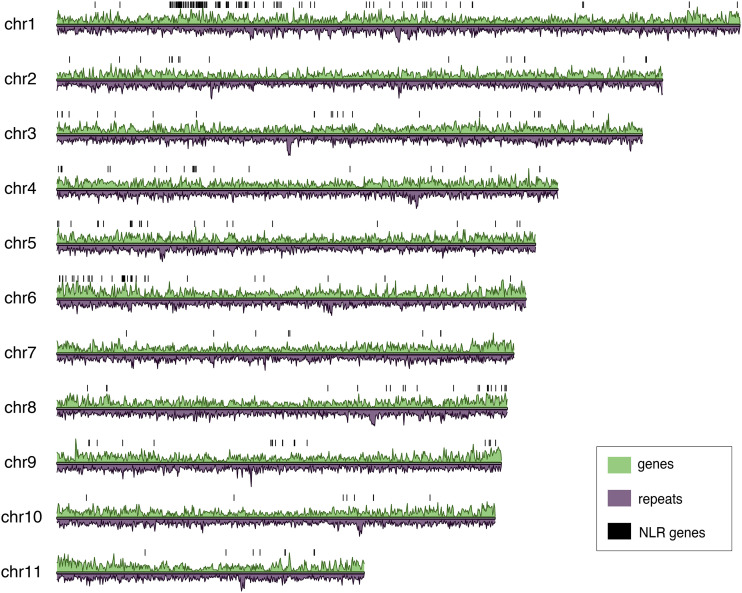
Repeat and gene density of giant sequoia 2.0. Gene density shown in green, repeat density shown in purple, both plotted in 1Mb windows. Locations of the consensus NLR genes indicated by black bars.

Distribution of the high-quality gene models spanned the length of all 11 chromosomes (Figure 2). Repeat density varied across the chromosomes, including overlap with annotated regions.

#### Assessing annotation completeness:

Completeness of the annotation was assessed with BUSCO (Table 3). The independent transcriptome completeness of 79% represents the maximum possible BUSCO score for the gene model sets. The BUSCO completeness of the final high-quality gene set was 53%, comparable to the same metric in *Pinus taeda* (53%, Wegrzyn *et al.* 2014) and *Pinus lambertiana* (50%, Stevens *et al.* 2016), suggesting the annotation of giant sequoia is on par with other conifer genomes.

#### Comparison to existing gymnosperm annotations:

While the genome size of giant sequoia is rather small for a gymnosperm (Table 4), it is consistent with both the genome size of other Cupressaceous conifers. Moreover, the identified repeat content of giant sequoia 2.0 (79%) is in line with observations from other gymnosperm taxa. The number of high quality annotated genes (41,632) is higher than many gymnosperm assemblies, though there is substantial variation in annotation results across the lineage. Average CDS length and average intron length in giant sequoia 2.0 fall within the observed ranges for existing assemblies, though notably the longest intron reported here is ∼1.4 Mb, nearly 400kb longer than the previous longest intron (from *Pinus taeda*, at over 800 kbp). That giant sequoia 2.0 contains an even longer intron is likely due to the contiguity of our assembly, which is unprecedented in conifers.

#### Orthology assignment and gene family evolution:

Using unigene sets from TreeGenes, twenty gymnosperm taxa passed the 60% threshold for BUSCO completeness (Supplementary Table S4). Orthogroup clustering of 697,337 protein sequences from these twenty gymnosperms plus an outgroup (*Amborella trichopoda*) yielded a total of 44,827 orthogroups (Supplementary Table S5). Only 196 were single-copy in all species, and 5,947 orthogroups had representatives from each species. Overall, 6.5% of all protein sequences were in species-specific orthogroups. Of the species-specific orthogroups (12,145 in total), 653 were unique to giant sequoia (Supplementary Table S6). Among the 653 giant sequoia-specific orthogroups, 599 were functionally annotated with either gene family assignment (367) sequence similarity search (6) or both (226) (Supplementary Table S4).

**Figure 3:**
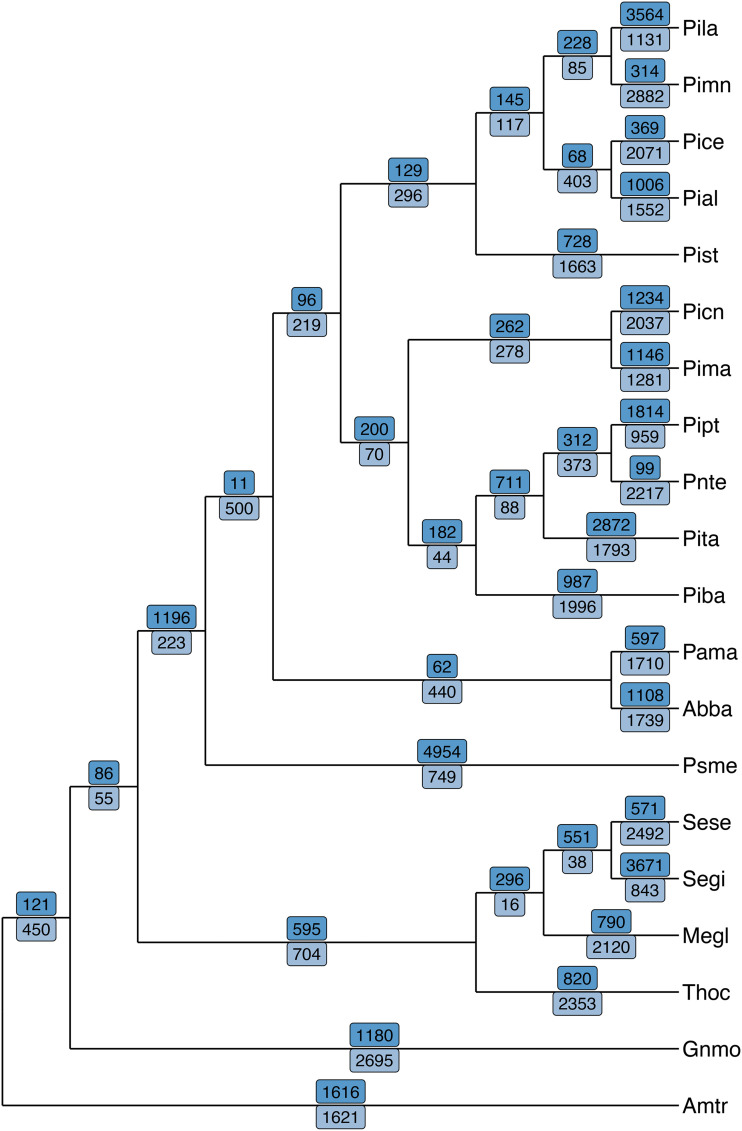
Gene family evolution along a gymnosperm cladogram. Numbers of expanded (bright blue, above branches) and contracted (light blue, below branches) orthogroups indicated in along each branch. Giant sequoia (Segi) experienced an overall expansion, with 3,671 orthogroups expanding and 843 contracting.

Orthogroup assignments were used as branch labels on a rooted species tree to show gene family contraction and expansion. On branch is the number of families that experienced expansion (dark blue, above) or contraction (light blue, below) (see Figure 3). Giant sequoia (Segi) experienced an overall expansion, with 3,671 families expanding and 843 families contracting since the species last shared common ancestor with coast redwood (*Sequoia sempervirens*; Sese).

**Figure 4:**
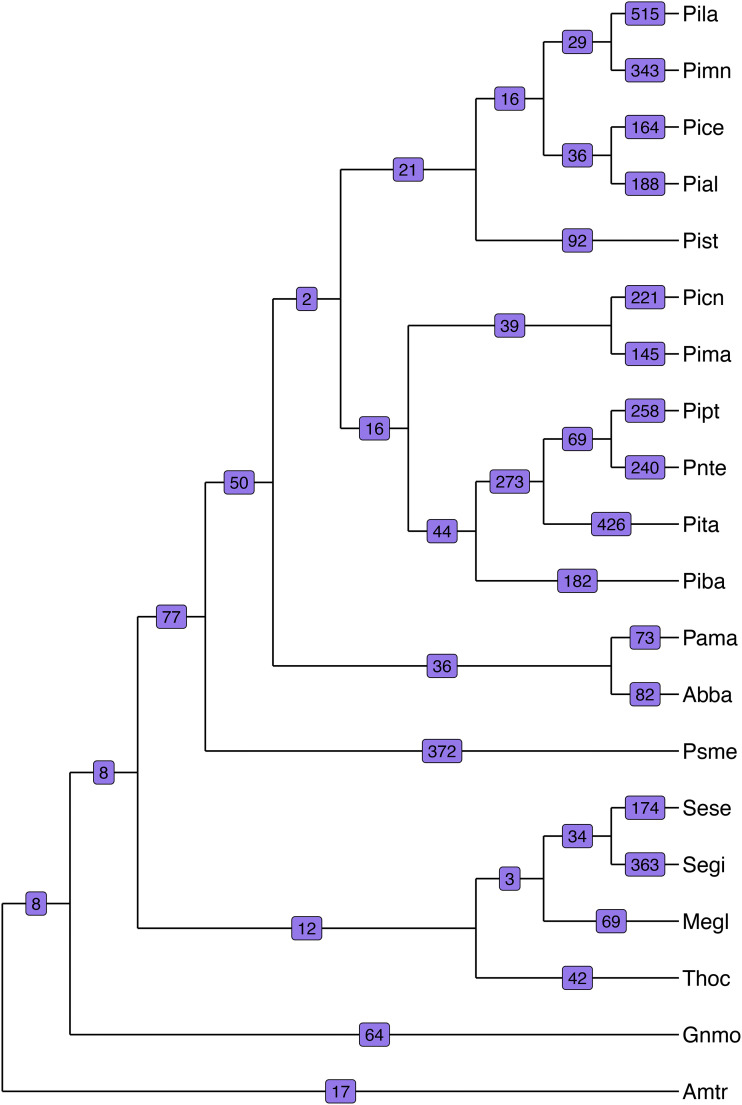
Rapid evolution along a gymnosperm cladogram. Numbers on each branch indicate the number of rapidly evolving gene families. Giant sequoia (Segi) has experienced rapid evolution in 363 gene families.

The expansions and contractions were further examined to identify nodes that experienced particularly rapid evolution. Many representatives of the Pinaceae have hundreds of gene families that experienced rapid change in size since their lineages diverged (Figure 4). Along the branch to giant sequoia (Segi), 363 orthologous groups rapidly expanded or contracted. The majority of these 363 orthogroups are moderately represented in the giant sequoia dataset (*e.g.*, with two to four members in an orthogroup), while others contain dozens of paralogs, up to over a hundred orthogroup members. Extracting the longest sequence from each of these yielded functional annotation with EnTAP for the rapidly evolving orthogroups. Rapidly expanding families were associated with primarily metabolic processes (GO:0090304, GO:0006796, GO:0044267) and macromolecule synthesis (GO:0009059, GO:0034645), in addition to molecular functions including metal-ion binding (GO:0046872), purine nucleotide (GO:0017076) and nucleoside (GO:0001883) binding, and kinase activity (GO:0016301). Rapidly contracting families were associated with biological processes such as protein (GO:0036211) and macromolecule modification (GO:0043412

and metabolic processes (GO:0044267, GO:0006796), and molecular functions including purine binding with nucleotides (GO:0017076) and nucleosides (GO:0001883), and phosphotransferase activity (GO:0016773).

#### NLR genes in the giant sequoia genome:

NLR proteins are structurally modular, typically containing an N-terminal coiled-coil (CC) domain, a Toll/interleukin-1 receptor (TIR) domain, or more rarely an RPW8-like CC domain; a conserved nucleotide binding domain (NB-ARC); and a C-terminal region comprising a variable number of leucine-rich repeats (LRRs) (Monteiro and Nishimura 2018). NLR genes in giant sequoia 2.0 were identified by first running the genomic sequence through the NLR-Annotator pipeline (Steuernagel *et al.* 2018). Importantly, this pipeline does not require masking of repetitive regions and does not rely on gene model predictions. NLR-Annotator outputs are categorized as either ‘complete’ or ‘partial’ depending on whether all canonical domains (CC/TIR, NB-ARC, LRR) are present, and then further categorized as ‘pseudo-’ if a stop codon is predicted in any domain. All categorizations should be considered tentative because the NLR-Annotator algorithm does not take intron/exon boundaries into account.

A total of 984 NLR genes were predicted by NLR-Annotator, of which 442 were identified as complete, 332 complete pseudo-, 88 partial, and 122 partial pseudo-. Of the 984, 712 included intact NB-ARC domains with fewer than 50% gaps in the alignment. This number is roughly twice the number of NLR genes found in cultivated rice (Zhou *et al.* 2004; Read *et al.* 2020) and is consistent with other conifers (Van Ghelder *et al.* 2019). NLR-gene coordinates of all NLR gene sequences in giant sequioa 2.0, and the relationships of the 712 based on an NB-ARC domain maximum likelihood tree are included in Supplementary Tables S8, S9, and S10 as well as Supplementary Figure S3.

**Figure 5:**
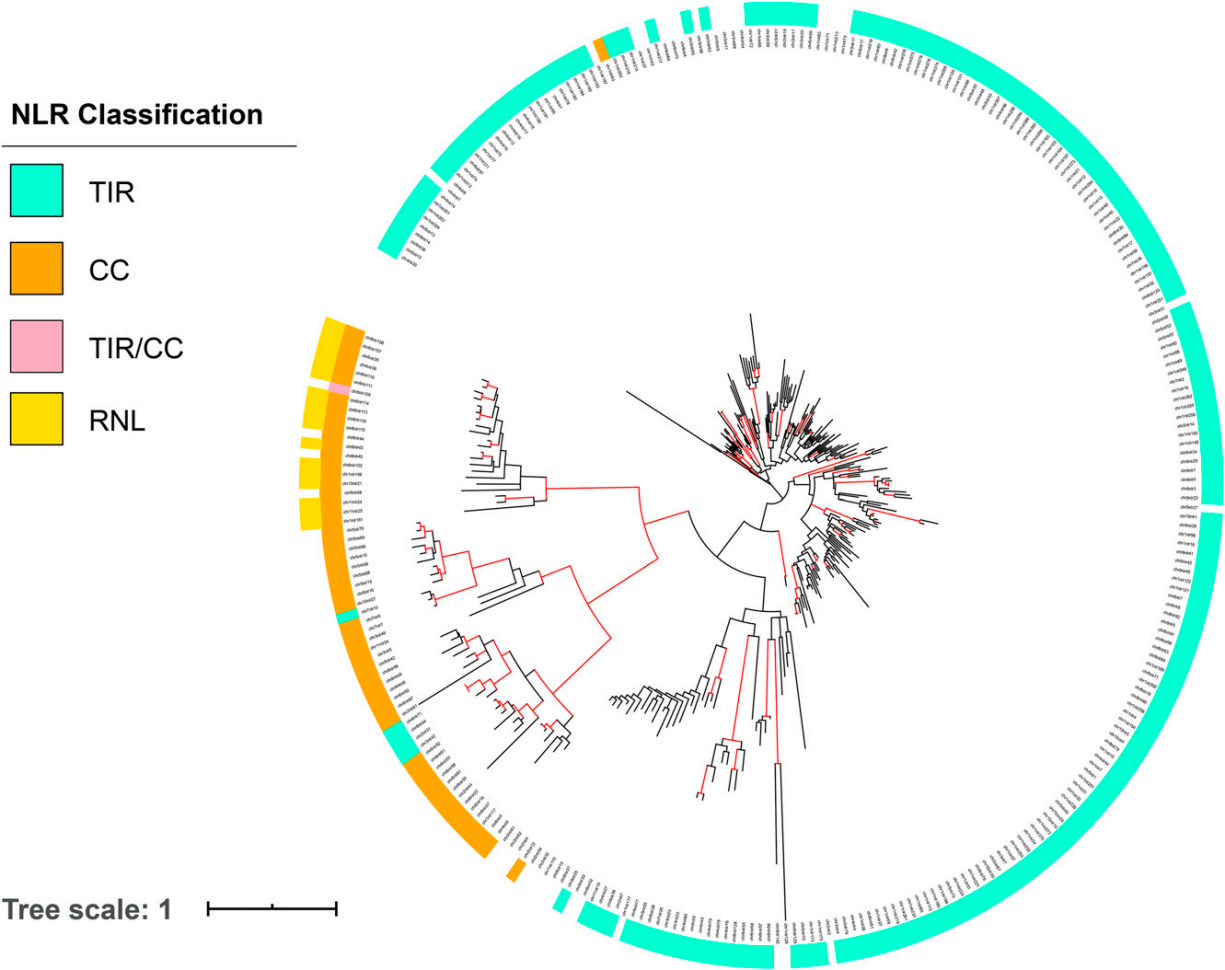
Maximum likelihood tree of encoded NB-ARC domains of the 300 consensus NLRgenes detected in the giant sequoia 2.0 assembly. Red branches indicate bootstrap support greater than 80%. The inner ring indicates predicted N-terminal TIR (blue) or CC (orange) domains. One of the 300 NLR contains motifs present in TIR and CC NLR proteins (pink). The outer ring indicates presence of an RPW8 motif present in the RNL sub-group of CC-NLRs. Tree is available at: http://itol.embl.de/shared/acr242

NLR-Annotator identifies all suspected NLR motif-encoding regions of the genome. This likely includes actual pseudogenes or gene fragments, both of which are important from an evolutionary perspective, but do not reflect the functional NLR arsenal. The NLR-Annotator output was cross-referenced with the giant sequoia genome annotation to identify the NLR genes that are supported by the annotation and therefore likely part of this arsenal; we refer to these 375 genes as consensus NLR genes. These NLR genes are found unevenly distributed across all 11 chromosomes, with the highest concentration on chromosome 1 (Figure 2). Of the 375, 256 were categorized by NLR-Annotator as complete, 71 as complete pseudo-, 35 as partial, and 13 as partial pseudo-. There were five cases in which two NLR-Annotator predicted NLRs overlapped a single annotated gene. In these cases, only one predicted NLR was included in analyses. Three hundred of the 375 consensus NLR genes encode NB-ARC domains that met our criteria (see Methods); a maximum likelihood tree was generated using these domains (Figure 5). Coordinates of the genes and their NB-ARC sequences are included in Supplementary Tables S5 and S7. NLR-Annotator predicted, non-consensus NLR genes may represent genes missed by the annotation, pseudogenes, or false positives.

To investigate the evolution of NLR genes in giant sequoia, the list of consensus NLR genes was compared with orthogroup assignments. Overall, consensus NLR genes were members of 63 orthogroups. Two of these 63 were orthogroups found to have experienced rapid expansion along the giant sequoia lineage. A study of NLR genes in limber pine (*Pinus flexilis*) similarly found duplication across all classes of NLR genes in that lineage (Liu *et al.* 2019), with NLR expansions attributed to both small-scale duplication and whole genome duplications alike. Given the demonstrated positive roles of NLR genes in resistance to pathogens and pests, and the contrasting role they may play in drought tolerance based on the observed downregulation of NLR genes under drought stress in several conifer species (Van Ghelder *et al.* 2019), additional examination of giant sequoia NLR genes may facilitate an understanding of the adaptive genetic landscape in this restricted species.

## Summary and Conclusions

The high quality of this assembly demonstrates the value of combining multiple sequencing technologies and leveraging a unique biological feature of conifers (sufficient haploid megagametophyte tissue for sequencing), along with the value of incorporating chromosome-conformation capture libraries to allow improvements in scaffolding. The giant sequoia genome assembly presented here provides a robust foundation for ongoing genomic studies to identify groves with evidence of local adaptation, with a focus on not only NLR genes but the many other genes and gene families potentially useful in conservation and management.

For the future, inferences about the evolutionary trajectory of conifers (and gymnosperms) will require a broadening of taxonomic focus. As the vast majority of conifer genomic research is centered on Pinaceae, developing resources in understudied conifer families is essential for meaningful comparative genomic work that could further inform conservation and management for iconic species.
